# Robust Template Adjustment Siamese Network for Object Visual Tracking

**DOI:** 10.3390/s21041466

**Published:** 2021-02-20

**Authors:** Chuanming Tang, Peng Qin, Jianlin Zhang

**Affiliations:** 1 Key Laboratory of Optical Engineering, Chinese Academy of Sciences, Chengdu 610200, China; tangchuanming19@mails.ucas.ac.cn; 2 Institute of Optics and Electronics, Chinese Academy of Sciences, Chengdu 610200, China; qinpeng191@mails.ucas.ac.cn; 3 School of Electronic, Electrical and Communication Engineering, University of Chinese Academy of Sciences, Beijing 100049, China

**Keywords:** visual tracking, siamese network, template adjustment, anchor-free regression, classification labels

## Abstract

Most of the existing trackers address the visual tracking problem by extracting an appearance template from the first frame, which is used to localize the target in the current frame. Unfortunately, they typically face the model degeneration challenge, which easily results in model drift and target loss. To address this issue, a novel Template Adjustment Siamese Network (TA-Siam) is proposed in this paper. The proposed framework TA-Siam consists of two simple subnetworks: The template adjustment subnetwork for feature extraction and the classification-regression subnetwork for bounding box prediction. The template adjustment module adaptively uses the feature of subsequent frames to adjust the current template. It makes the template adapt to the target appearance variation of long-term sequence and effectively overcomes model drift problem of Siamese networks. In order to reduce classification errors, the rhombus labels are proposed in our TA-Siam. For more efficient learning and faster convergence, our proposed tracker uses a more effective regression loss in the training process. Extensive experiments and comparisons with trackers are conducted on the challenging benchmarks including VOT2016, VOT2018, OTB50, OTB100, GOT-10K, and LaSOT. Our TA-Siam achieves state-of-the-art performance at the speed of 45 FPS.

## 1. Introduction

As one of the fundamental tasks in the field of computer vision, visual object tracking is widely used in many fields such as intelligent surveillance, human-machine interaction, unmanned vehicles, and so on. It aims to continuously track the target in subsequent frames by giving only its location in the first frame.

There are two paradigms for visual tracking: Siamese network tracking methods and tracking-by-detection methods [1]. Siamese network tracking is the most popular method currently due to its balance performance on accuracy and speed. It regards the tracking challenge as a problem of matching the candidate target area in the current frame with the template. SiamFC [2] is the ground-break work around the Siamese network, while SiamRPN [3] is the inspiring and classic work of multi-branches trackers. It employs the region proposal network [4] to divide the prediction subnetwork into classification branch and regression branch. By jointly training the two branches cunningly, SiamRPN avoids extracting multi-scale feature maps for the object scale change. After that, a large number of anchor-based Siamese trackers were proposed. However, it is difficult for these trackers to refine the anchors whose overlap with the target objects is small. This will cause tracking failure when the classification results are not reliable [5].

The input of the Siamese network generally includes the object template and the scene frame. The object template is usually initialized in the first frame and kept fixed in the following frames [1]. However, when the object encounters dramatic appearance deformation, illumination variations, and big rotation changes, the object features will be difficult to match with those of the initial template. The feature change over time is striking, and it makes the initial template degraded heavily. Hence, an appropriate tracking template update method is essentially necessary.

**Figure 1:**
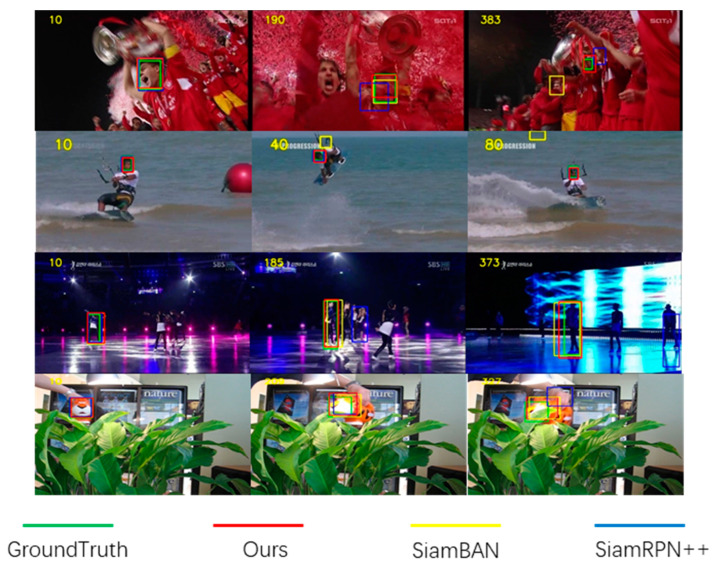
Visual comparison of TA-Siam with state-of-the-art trackers on four video sequences: soccer, kitesurf, skating1, motor-rolling. TA-Siam expresses our proposed Template Adjustment Siamese Network tracker. The number in the top left corner of each image represents the video frame number. Separately, these sequences represent four different challenges, respectively. Compared with SiamBAN [6] and SiamRPN++ [7], our proposed tracker can avoid bounding box drift and object loss phenomenon.

To address these issues, we propose a robust Template Adjustment Siamese Network (TA-Siam). TA-Siam has three input branches (i.e. a new branch called template adjustment branch is designed besides the conventional two branches). The template adjustment branch makes the subsequent frames as input, and extracts and fuses new frame’s feature into the feature of previous template. What is more, our adjustment strategy can be used as a plug-and-play module for other Siamese trackers. Figure 1 shows the visual comparison of different trackers.

In general, the main contributions of this work can be summarized as:

- A plug-and-play template adjustment Siamese network is designed for visual tracking, which sharply reduces the risk of model drift and object loss;
- In classification and regression branches, the rhombus labels and anchor-free strategy are presented to accurately infer the center point and sides of the bounding box. In the training phase, the Distance-Intersection over Union (D-IOU) loss is realized to train the anchor-free regression subnetwork.
- Our proposed tracker achieves state-of-the-art tracking performance with an average speed of 45 FPS (Frames Per Second) on six challenge benchmarks, including VOT2016 [8], VOT2018 [9], OTB50 [10], OTB100 [11], GOT-10k [12], and LaSOT [13].

The remaining contents of this paper are organized as follows. We briefly review some related works of visual tracking in Section 2. In Section 3, our proposed tracking method is explained in detail. In Section 4, our method TA-Siam is evaluated and analyzed on five challenging benchmarks. In Section 5, the whole paper is summarized briefly.

## 2. Related Work

### 2.1. Siamese Network

The pioneering works of Siamese network trackers are SINT [14] and SiamFC [2], which regard the tracking problem as the similarity measurement of the target between the first frame and current frame. SiamRPN [3] draws on the Region Proposal Network (RPN) to get more various scale ratio bounding boxes. SiamRPN++ [7] breaks through the depth of convolution layers in the tracking field for the first time. It obtains excellent feature extraction ability with a 50-layer depth residual network [15] and eliminates the center deviation by randomly changing the target position in training process. The Siamese network has achieved advanced performance recently, but its performance, especially robustness, is still worse than the online update state-of-the-art trackers, including ATOM [16], DiMP [17], and PrDiMP [18].

### 2.2. Template Updating

Template updating trackers are mainly used in the correlation filtering framework. Joint representation and truncated inference learning for correlation filter-based tracking [19] employs a long short-term memory to estimate the current template by storing previous templates in memory during inference phase, which is highly computing complex and time-consuming. Real-time visual tracking by deep reinforced decision making [20] uses reinforcement learning to store templates and select one as a template while tracking. However, it fails to combine target features from multiple frames, and it is hard to find missing targets. 

Siamese trackers typically do not update the initial template due to background noise and tracking speed. However, complete reliance on the initial template will caused catastrophic drift and tracking failure over time. To adapt to the target appearance variations, the initial template through regularized linear regression are proposed in the Fourier domain by DSiam [21]. Only considering the initial template transformation makes it ignore the subsequent template variations, which makes it ignore the historical target variations. On the contrary, our work not only uses the first frame but also uses the information of the subsequent frames to adjust the template and accumulate the target variations. In order to adapt to the changing environment of object and keep the template robustly to multiple scenes, some correlation filter trackers implement a linear update strategy frame by frame [22,23]. However, the frame-by-frame updating strategy is not essential and will heavily affect the speed of the tracker. UpdateNet [1] employed a simple convolutional neural network to update the template, which aims to estimate the optimal template for the next frame based on the given initial template, the accumulated template, and the template of the current frame. However, over time, updating the template throughout the video sequence will still contaminate the template with error accumulation.

### 2.3. Anchor-Free Regression

Different from anchor-based regression, anchor-free regression directly predicts the location of objects. It avoids hyper-parameters brought by the anchor boxes, making it more flexible and lightweight. Inspired by the popularity of anchor-free detectors [24,25,26], some anchor-free trackers arise nowadays. 

Inspired by FCOS [26] in object detection, SiamFC++ [27] borrows regression branch of FCOS and adds a center-ness branch to increase the weight of the center. SiamBAN [6] employs ellipse labels to improve the classification branch performance. While SiamCAR [28] changes the basic Siamese network structure and carries out multi-layer fusion before correlation. The anchor-free regression in our method is inspired by SiamBAN but is different from SiamBAN. It will be discussed in detail in Section 3.2. 

Cross-entropy loss is the general loss function of classification subnetwork in Siamese trackers [3,6,7,28], while SiamFC++ uses the focal loss [29] to pay attention to the positive sample. As for the regression loss functions, trackers like SiamRPN and SiamRPN++ use smooth L1 loss with normalized coordinates. Others [5,6,27,28] adopt the Intersection Over Union (IOU) [30] loss to maximize the overlap rate between the prediction box and ground truth box. Nonetheless, if the prediction box and ground truth box do not intersect, then IOU will degenerate to zero. Hence, IOU loss cannot work well when the overlap rate of the bounding boxes is close to zero. To solve this problem, our work employs a new distance-IOU loss [31] for anchor-free works (Section 3.3).

## 3. Methods

In this section, we will introduce our TA-Siam tracker in detail. As mentioned earlier, the main structure of the proposed method is three input branches and classification-regression subnetworks. We adopted the advanced tracker SiamBAN [6] as our baseline method. SiamBAN uses ResNet-50 [15] as the backbone network and creates the ellipse label for classification. However, with the initial template used in all video sequences, the template degradation makes it easy to lose the target. 

**Figure 2:**
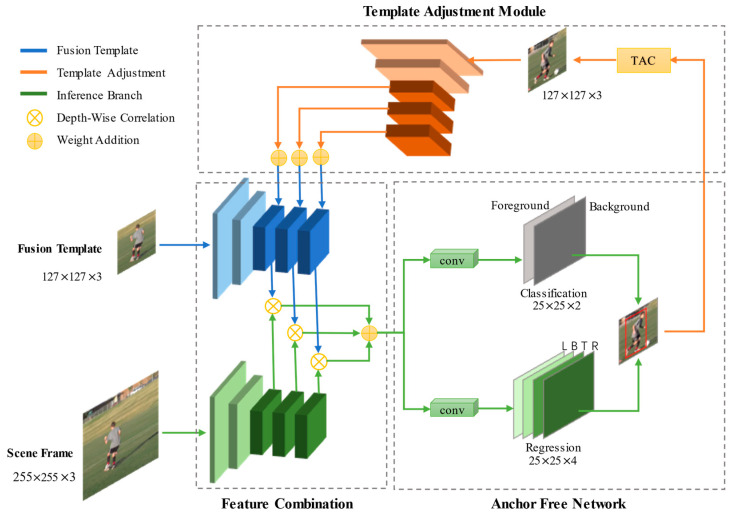
The overview of our proposed TA-Siam framework. TAC means Template Adjustment Controller. The template adjustment module has the characteristics of plug-and-play.

Figure 2 shows the framework of our TA-Siam. It adopts a stable template adjustment strategy to maintain the reference of the target template and the matching degree with the target. Furthermore, compared with SiamBAN, TA-Siam has more adaptive classification labels and superior training efficiency. It enabled our tracker to robustly deal with hard scenes such as in-plane rotation, illumination variation, out of view, and occlusion.

### 3.1. Template Extraction and Adjustment

Following the basic architecture of our baseline tracker, we employ the ResNet-50 network as our backbone for feature extraction. ResNet-50 contains five convolutional blocks, labeled $\varphi_{1}$, $\varphi_{2}$, $\varphi_{3}$, $\varphi_{4}$, $\varphi_{5}$. The spatial resolution of each block decreases in turn. In order to take advantage of shallow and deep features, we take $\varphi_{3}$, $\varphi_{4}$, and $\varphi_{5}$ as multi-output. To facilitate the subsequent fusion of multi-level prediction, we delete the down sampling operation from $\varphi_{4}$ and $\varphi_{5}$ to keep the same spatial resolution. 

The framework backbone consists of three branches. The first one, called the template adjustment branch, takes the output of the template adjustment controller $T_{i}$ as input. It is composed of the object area obtained from previous K-frame tracking. The second one is a fusion template branch ${\tilde{T}}_{i}$. It fuses the previous fusion template with the current adjusted template to obtain a new tracking template, and the fusion template will be made as a matching template saved in memory. The third one, called the inference branch, takes the scene $S_{i}$ as the input image. The three branches share the parameters completely. The output of the scene $S_{i}$ and fusion template branch ${\tilde{T}}_{i}$ have identical structure in feature extraction:

(1) $$\begin{array}{l} F_{k}\left(X\right)=\varphi_{k}\left(S_{i}\right)\\ F_{k}\left(Z\right)=\varphi_{k}\left({\tilde{T}}_{i}\right) \end{array}$$

where *k* is the index number of convolution blocks, which should be taken as 3, 4, and 5.

Template adjustment mainly affects the template matching stage, which makes the network more sensitive to the proposed bounding boxes with background changes. As an independent branch, an appropriate Template Adjustment Controller (TAC) is designed to determine whether to adjust the frame. The TAC module can be defined as follows:

(2) $$\delta =\{\begin{array}{c} 1\\ 0 \end{array}\begin{array}{c} S_{best}>\alpha \;\mathrm{and}\;T_{i}-T_{i-1}=f\\ otherwise \end{array}$$

where α is the confidence score threshold, which ensures to screen high confidence templates and avoid excessive background noise pollution. f is the interval between the last adjustment video frame and the current frame, which can help reduce the computation and keep real-time tracking speed. Functionally, TAC maintains the adjustment frequency and improves the quality of template to prevent template degradation. 

The template features are extracted layer-by-layer before fusion. Specifically, the process of the template adjustment module (TAM) is as follows:

(3) $$F_{k}\left({\tilde{T}}_{i}\right)=\lambda \delta \varphi_{k}\left(T_{i}\right)+{\left(1-\lambda \right)}^{\delta }\varphi_{k}\left(T_{i-1}\right)$$

where λ is the template fusion weight parameter. While in the correlation layer, Depth-Wise Correlation (DW-Corr) [7] can be expressed as:

(4) $$R_{k}=F_{k}\left(X\right)*F_{k}\left(Z\right)$$

In order to make full use of multi-level features, a weighted addition feature map can be formulated as:

(5) $$\begin{array}{l} R_{cls}=\sum_{k=3}^{5}\alpha_{k}R_{k}\\ R_{reg}=\sum_{k=3}^{5}\beta_{k}R_{k} \end{array}$$

where $R_{cls}$ is the classification feature map, $R_{reg}$ is the regression feature map, $\alpha_{k}$ and $\beta_{k}$ are hyperparameters of corresponding weights.

### 3.2. Classification Label Selection and Anchor-Free Regression

In the classification branch, rectangle boxes are the general sample labels for different trackers training [3,7,28]. It makes the tracker distracted by every rectangle pixel, which weakens the center attention and classification performance. Compared with rectangular labels, ellipse labels can reduce the error classification ratio, and achieve more robust classification performance by setting a buffer. Inspired by this, we propose the novel rhombus labels, which are more stable than ellipse [6] and rectangles [7], to abate the probability of error classification. It takes full account of the target scale, aspect ratio, and shape. In addition, the buffer area between the rhombuses is more than the ellipses and rectangles, making more target edges fall in the buffer.

**Figure 3:**
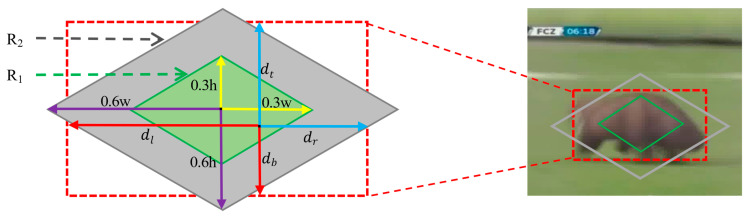
The rhombus classification labels and four sides regression of bounding box.

The center point, width, and height of the ground-truth box are represented by $\left(x_{c},y_{c}\right),w,h$. Rhombus labels can be formulated as:

(6) $$\gamma_{1}\left|p_{x}-x_{c}\right|+\gamma_{2}\left|p_{y}-y_{c}\right|=\frac{\gamma_{1}\gamma_{2}}{2}$$

where $\gamma_{1}$ and $\gamma_{2}$ are the adaptive scale parameters to adjust the aspect ratio and target scale. As shown in Figure 3, with $\left(x_{c},y_{c}\right)$ as the center, 0.3w and 0.3h are the scale parameters, the rhombus $R_{1}\left(p_{x},p_{y}\right)$ can be represented as:

(7) $$0.3w\left|p_{x}-x_{c}\right|+0.3h\left|p_{y}-y_{c}\right|-0.045wh=0$$

With $\left(x_{c},y_{c}\right)$ as the center, 0.6w and 0.6h are the scale parameters, the rhombus $R_{2}\left(p_{x},p_{y}\right)$ can be represented as:

(8) $$0.6w\left|p_{x}-x_{c}\right|+0.6h\left|p_{y}-y_{c}\right|-0.18wh=0$$

Intuitively, our rhombus labels take more attention to the center than rectangles. Furthermore, the rhombus labels make more error-prone samples placed in the transition zone. The sample point $\left(p_{x},p_{y}\right)$ label can be defined as:

(9) $$label=\{\begin{array}{c} 1\\ 0\\ -1 \end{array}\begin{array}{c} if\;R_{1}\left(p_{x},p_{y}\right)<0\\ if\;R_{2}\left(p_{x},p_{y}\right)>0\\ otherwise \end{array}$$

where the label will be set as positive when the point falls inside the rhombus $R_{1}$, and it will be set as negative when the point falls outside the rhombus $R_{2}$, and it will be ignored when the point falls between the rhombus $R_{1}$ and $R_{2}$.

In the regression branch, the object target is represented as:

(10) $$\begin{array}{l} d_{l}=p_{x}-x_{0}\\ d_{t}=p_{y}-y_{0}\\ d_{r}=x_{1}-p_{x}\\ d_{b}=y_{1}-p_{y} \end{array}$$

where $\left(x_{0},y_{0}\right)$ and $\left(x_{1},y_{1}\right)$ represent the ground-truth corner points of top-left and bottom-right, respectively. $d_{l},d_{t},d_{r},d_{b}$ represent the distance from $\left(p_{x},p_{y}\right)$ to the left, top, right, and bottom bounding box, respectively, as shown in Figure 3.

### 3.3. Loss Function with Distance Constraint

**Figure 4:**
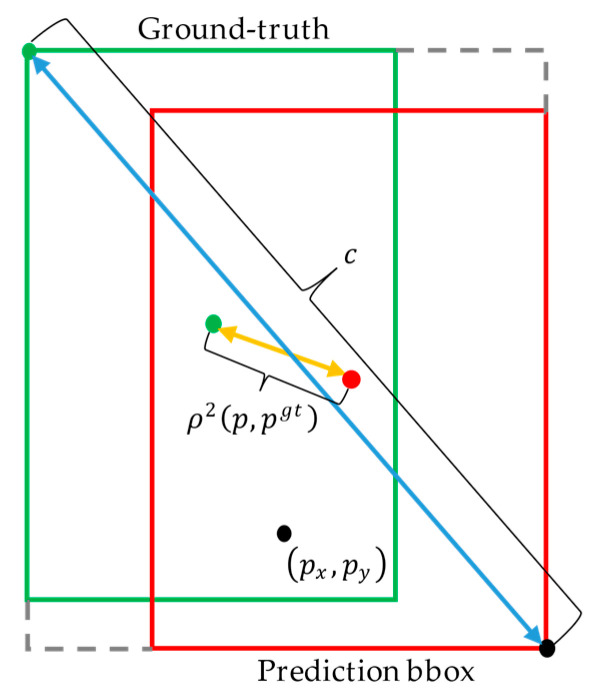
The distance of central points and diagonal points.

In our proposed work, the distance-IOU loss [31] is applied for regression in the training process instead of IOU loss. Distance-IOU add distance constraint on the basis of the overlap area. On the whole, the regression loss can be formulated as:

(11) $$L_{reg}=1-IOU+\frac{\rho^{2}\left(p,p^{gt}\right)}{c^{2}}$$

where p is the center of the prediction bounding box and $p^{gt}$ is the center of the ground-truth box. $\rho^{2}\left(p,p^{gt}\right)$ denotes the Euclidean distance between the central points of prediction bounding box and ground-truth. c represents the distance between the farthest corners of two boxes, as shown in Figure 4. While in the specific calculation method, it has great distinction with the anchor-based network.

While $g_{l},g_{t},g_{r},g_{b}$ represent the distance from four sides of the ground-truth box to the point $\left(p_{x},p_{y}\right)$. $c^{2}$ can be calculated in details:

(12) $$\begin{array}{c} w_{u}=\max\left(g_{l},d_{l}\right)+\max\left(g_{r},d_{r}\right)\\ h_{u}=\max\left(g_{b},d_{b}\right)+\max\left(g_{t},d_{t}\right)\\ c^{2}=w_{u}{}^{2}+h_{u}{}^{2} \end{array}$$

where $w_{u}$ and $h_{u}$ represent the width and height of the union between the prediction and ground-truth box. The width $w_{i}$ and height $h_{i}$ of the intersection between the prediction and ground-truth box can be calculated as follows:

(13) $$\begin{array}{c} w_{i}=\min\left(g_{l},d_{l}\right)+\min\left(g_{r},d_{r}\right)\\ h_{i}=\min\left(g_{b},d_{b}\right)+\min\left(g_{t},d_{t}\right) \end{array}$$

$\rho^{2}\left(p,p^{gt}\right)$ can be calculated in details:

(14) $$\begin{array}{c} w_{c}=\frac{\left(g_{l}+g_{r}+d_{l}+d_{r}\right)}{2}-w_{i}\\ h_{c}=\frac{\left(g_{t}+g_{b}+d_{t}+d_{b}\right)}{2}-w_{i}\\ \rho^{2}\left(p,p^{gt}\right)=w_{c}{}^{2}+h_{c}{}^{2} \end{array}$$

where $w_{c}$ and $h_{c}$ are the width and height of the center point between the ground-truth and prediction bounding box.

Meanwhile, the classification subnetwork is optimized by minimizing the cross-entropy loss:

(15) $$L_{cls}=-\left[y\log y^{\prime }\left(1-y\right)\log\left(1-y^{\prime }\right)\right]$$

The joint training of anchor-free regression and classification subnetwork optimize the following multi-task loss function:

(16) $$L=\lambda_{1}L_{cls}+\lambda_{2}L_{reg}$$

where the hyperparameters $\lambda_{1}$ and $\lambda_{2}$ are the weights of classification and regression, respectively. We empirically set $\lambda_{1}=2$ and $\lambda_{2}=1$ while training.

## 4. Experiments

To evaluate the performance of our method, the comparing experiments with the State-Of-The-Art (SOTA) tracker and the ablation experiments were carried out on typical datasets in the section. Firstly, the implementation details are presented. Then the experiments of comparison of our method with the SOTA tracker are described, and experimental results are analyzed and interpreted in detail.

### 4.1. Implementation Details

During the training, 127×127 and 255×255 pixels were the sizes of input exemplar image and scene image, respectively. The backbone was initialized with the parameters pretrained on ImageNet [32], while other parts were randomly initialized. We trained our tracker with the data from ImageNet VID [32], COCO [33], YouTube-BB [34], Image DET [32], GOT-10k [12], and LaSOT [13]. It should be noted that both LaSOT and GOT-10k experiments were only trained on their own training sets, respectively. We trained a total of 25 epochs with Stochastic Gradient Descent (SGD). The batch size was 20, while the learning rate was different in epochs. During the first five epochs, the learning rate increased from 0.001 to 0.005, and then during the last 20 epochs it decayed from 0.005 to 0.00001. The weight decay and momentum were 0.0001 and 0.9, respectively. Empirically, the TAC hyperparameter α can be set from 0.95 to 0.99, f is generally set from 25 to 35 for keeping inference speed. The TAM hyperparameter λ can be set from 0.15 to 0.25. All experiments were carried out on a PC equipped with an Intel i5-9600KF 3.7GHz CPU, 16G memory, and Nvidia RTX 2060s GPU.

### 4.2. Comparison with State-of-the-Art

To evaluate the proposed method extensively, we compared TA-Siam with more than 30 recent SOTA trackers on six benchmark datasets, include VOT2016, VOT2018, OTB50, OTB100, GOT10k, and LaSOT.

#### 4.2.1. Results on VOT2016 and VOT2018

VOT challenge is a representative competition in the field of object tracking. The trackers are usually evaluated by the metrics Expected Average Overlap (EAO), robustness (average number of failures) and accuracy. Robustness measures how many times the tracker loses the target (fails) during tracking. Accuracy is the average overlap between the predicted and ground truth bounding boxes during successful tracking periods. 

**Figure 5:**
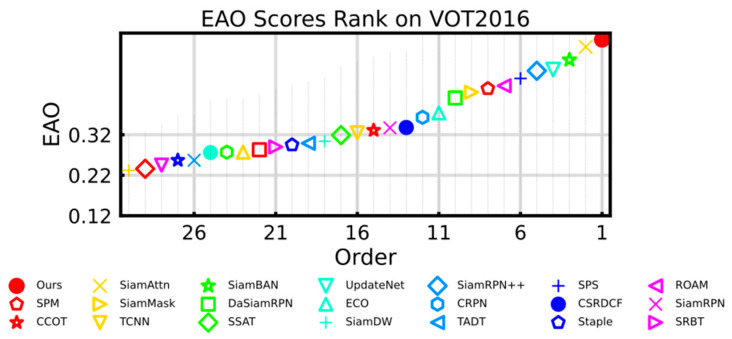
Expected Average Overlap (EAO) ranking of the evaluated tracker on VOT2016 benchmark.

We compared our tracker with 29 state-of-the-art trackers, the EAO score ranking of VOT2016 [8] is shown in Figure 5. Our method gets the best performance in EAO score compared to others. Specifically, TA-Siam was compared with SiamAttn [35], SiamBAN [6], UpdateNet [1], SiamRPN++ [7], SPS [36], ROAM [37], SPM [38], and SiamRPN [3] in detail. The performance comparison can be seen in Table 1. In the previous methods, SiamAttn achieved the state-of-the-art performance in all metrics, while our TA-Siam outperformed SiamAttn in EAO score and robustness. As for the comparison with our baseline tracker SiamBAN, our method improved the EAO by 6.1 points and reduces the failure rate by 5.1 points.

**Figure 6:**
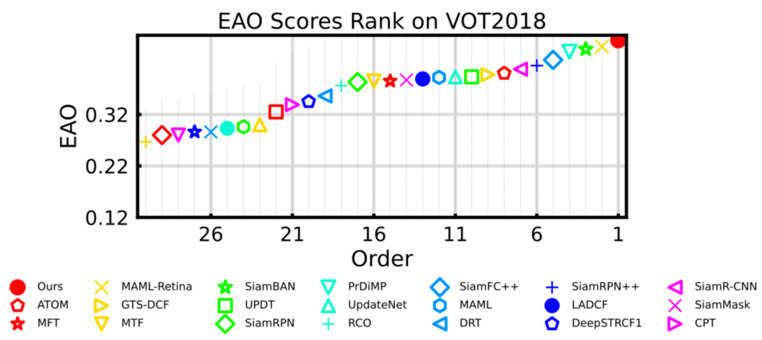
EAO ranking of the evaluated tracker on VOT2018 benchmark.

VOT2018 [9] replaces 10 challenging video sequences, adding more fast motion and similar target videos. As shown in Figure 6, our method achieved state-of-the-art performance in terms of EAO score. Details are compared in Table 2. The proposed tracker TA-Siam got the best score in EAO and robustness. Comparison by tracker framework, our TA-Siam achieved EAO improvements of 5.5 points compared with anchor-based tracker SiamRPN++. Compared with the online update SOTA trackers PrDiMP [18], the proposed method was superior in EAO and robustness. Compared with anchor-free trackers like SiamFC++ [27] and SiamBAN [6], we obtained similar accuracy but more advanced EAO and robustness performance. It is worth noting that the improvements of our trackers mainly come from the robustness score, which benefits from template adjustment.

**Figure 7:**
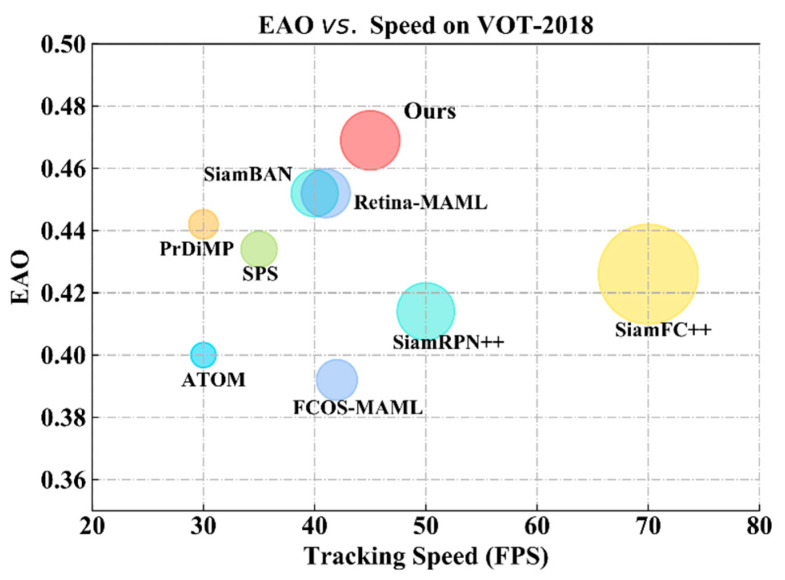
Comparisons of EAO and speed on VOT2018 benchmark.

In terms of tracking speed, we compared it with night state-of-the-art trackers including SiamBAN [6], PrDiMP [18], Retina-MAML [39], FCOS-MAML [39], SiamRPN++ [7], SiamFC++ [27], ATOM [16], and SPS [36]. Our TA-Siam achieves the advanced EAO score (0.469) while running at 45 FPS. It can be seen from Figure 7 that the speed of our tracker is faster than our baseline tracker SiamBAN and online updating trackers, such as PrDiMP, SPS, and ATOM. Compared with anchor-based SOTA tracker SiamRPN++ and anchor-free tracker SiamFC++, we maintained a real-time speed with much higher EAO score. Therefore, it indicates that our TA-Siam achieved a good balance between performance and tracking speed.

#### 4.2.2. Results on OTB50 and OTB100

**Figure 8:**
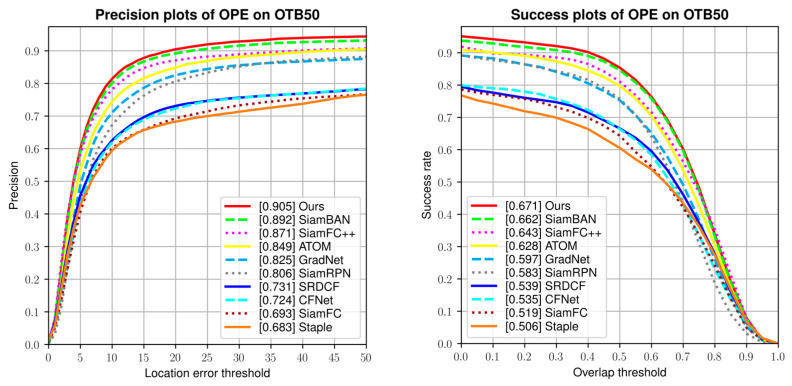
The precision plots and success plots on OTB50 dataset.

The OTB benchmarks consists of OTB50 [10] and OTB100 [11]. They contain 50 and 100 real-word video sequences separately. Besides, there are eleven challenge attributes to different sequences. Our TA-Siam was evaluated with the One-Pass Evaluation (OPE) method. Success rate and precision rate are the main metrics. Our TA-Siam was compared with the state-of-the-art trackers including SiamFC++ [27], ATOM [16], SiamBAN [6], SiamRPN [3], GradNet [40], CFNet [41], SRDCF [42], Staple [43], SiamFC [2], and other baseline trackers. While in OTB50 benchmark, TA-Siam achieves the SOTA performance in success rate and precision rate. As shown in Figure 8.

**Figure 9:**
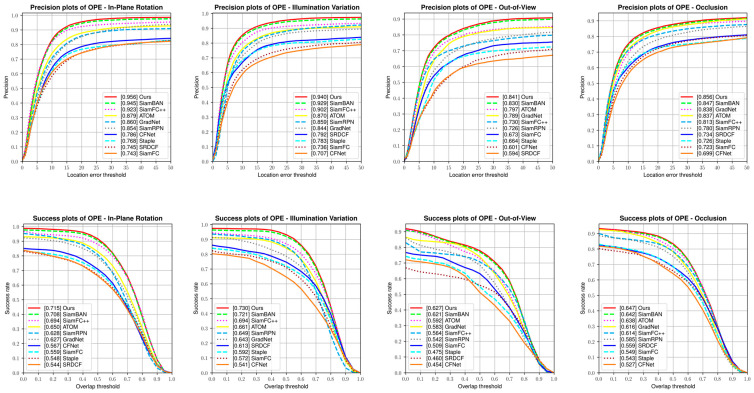
The evaluation on OTB100 dataset with four challenging attributes.

While in OTB100 benchmark, our tracker also acquired the best scores in both metrics. Especially, it significantly improved the tracking performance against the impacts of in-plane rotation, illumination variation, out of view, and occlusion challenges. As shown in Figure 9.

#### 4.2.3. Results on GOT-10k

**Figure 10:**
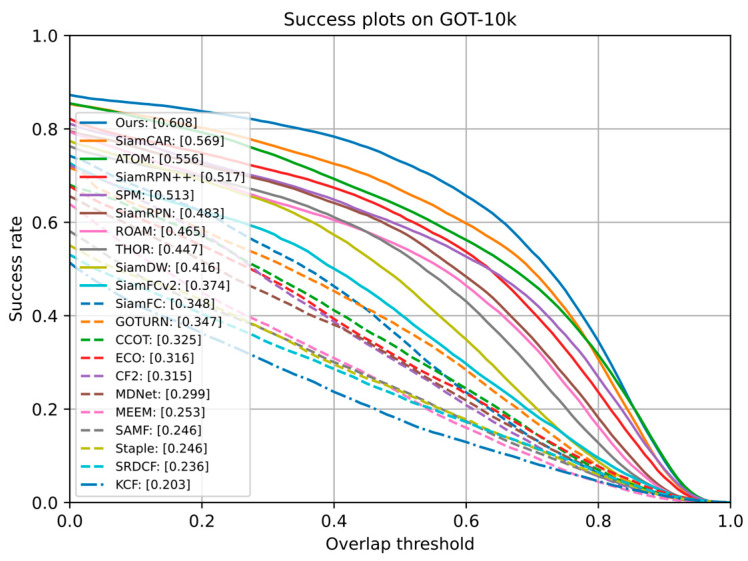
Comparison results on the GOT-10k benchmark.

GOT-10k [12] is a large-scale tracking benchmark with more than 10,000 videos. It uses the online server to evaluate the test subset which consists of 180 video sequences. The analysis results were automatically provided by the official website. We strictly complied with the benchmark protocols and trained our model with only GOT-10k training subset. The Average Overlap (AO) and Success Rate (SR) are the evaluate indexes of GOT-10k. The success rate has two thresholds (0.5 and 0.75). We evaluated our TA-Siam and compared it with 19 state-of-the-art and baseline trackers. The results of these trackers are all provided by the dataset official website. Figure 10 shows the success plots of 20 trackers. Our TA-Siam is significantly superior to other methods on GOT-10k.

Table 3 shows the details of some recent SOTA trackers performance, including SiamFC++ [27], D3S [44], SiamCAR [28], ATOM [16], SiamRPN++ [7], SPM [38], ROAM [37], THOR [45], and SiamDW [46]. TA-Siam outperforms all top-performing trackers in both AO and SR_0.5_ metrics. Compared with anchor-based tracker SiamRPN++, it improved by 18%, 19%, and 40%, respectively, in terms of AO, SR_0.5_, and SR_0.75_. Meanwhile, TA-Siam outperforms anchor-free trackers SiamCAR and SiamFC++ with significant margins. Moreover, our TA-Siam is superior to the recent segmentation tracker D3S in AO and SR_0.5_.

#### 4.2.4. Results on LaSOT

The Large-Scale Single Object Tracking (LaSOT) benchmark provides large-scale, high-quality dense annotations with 1400 videos in total [13]. It has 70 categories of objects, each containing twenty sequences, and the average video length is more than 2500 frames. Each sequence comprises various challenges deriving from the wild where target objects may disappear and re-appear again in the view. It is useful to evaluate the stable and long-term tracking ability of the trackers. 

**Figure 11:**
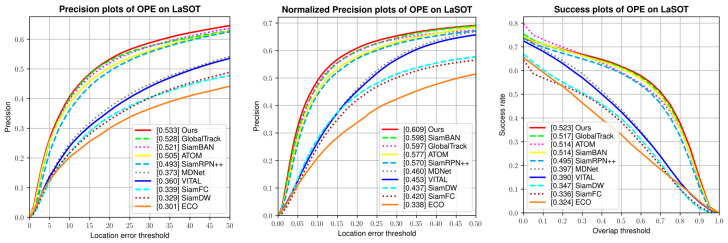
Comparison with other trackers on LaSOT test set in terms of the precision, normalized precision, and success plots.

Similar to OTB datasets, we evaluated our TA-Siam on the test set which consists of 280 videos in One-Pass Evaluation (OPE). The evaluation metrics are precision plot, normalized precision plot, and success plot. Our TA-Siam was compared with SOTA trackers including SiamBAN [6], GlobalTrack [47], SiamRPN++ [7], ATOM [16], and other baseline trackers, the results are illustrated in Figure 11. 

Compared with SiamBAN, our TA-Siam improves the scores by 1.2, 1.1, and 0.9 points, respectively, for the three metrics. While compared with the baseline approaches, our tracker improves by over 16%, 14.9%, and 12.6%, respectively. Notably, our tracker outperforms the recent online updating tracker ATOM by 2.8%, 3.2%, and 0.9% respectively. Meanwhile, our tracker also surpasses the SOTA long-term tracker GlobalTrack in all three metrics. From the above comparison, our TA-Siam indicated its powerful stability ability of long-term tracking. 

### 4.3. Ablation Study

In order to explore the efficacy of different components in our tracker, we performed a component-wise analysis on VOT2016, as shown in Table 4. The baseline tracker consists of anchor-free regression and a standard classification network. The baseline ① obtained an EAO of 0.494 and robustness of 0.158. In ②, after the distance-IOU loss was added in the anchor-free regression while training, the EAO increased 1.1 points. This indicates that it is advantageous for distance-IOU to obtain more approximate regression. Comparing ③ with ①, the template adjustment mechanism can bring a vast improvement in EAO, from 0.494 to 0.538. Meanwhile, the failure rate decreased from 0.158 to 0.121, indicating that template adjustment can abate the drift range of prediction bounding box and target lost drastically. For ④ and ③, combined with template adjustment and distance-IOU loss regression, TA-Siam improves 1.7 points on the basis of ③, indicating that distance-IOU loss is also significant in the template adjustment network. In general, we observe that every component in our framework is necessary and important. The combination of our template adjustment module and anchor-free distance-IOU loss achieved the state-of-the-art results in our experiments.

**Figure 12:**
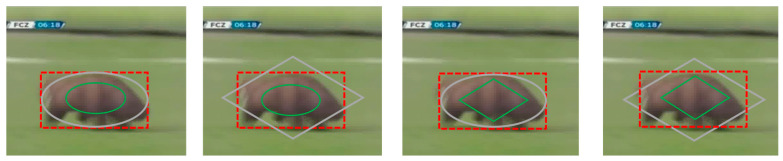
Four different sample label combinations: Ellipses, rhombus-ellipse, ellipse-rhombus, and rhombuses.

To confirm the validity of rhombus labels, we further compared the impact of different classification label shapes. The label combinations are shown in Figure 12. The baseline is the proposed tracker with our template adjustment strategy. While training, they all employ distance-IOU loss to accelerate convergence. We compared ellipse labels, ellipse-rhombus labels, rhombus-ellipse labels, and rhombuses labels on the baseline. To be fair, we kept the ellipse label parameters consistent with SiamBAN. The results are reported on GOT-10k in Table 5. They were all trained in the GOT-10k training set and tested in the GOT-10k test set with the same hyperparameters. While training, every epoch contains 600,000 pairs of pictures.

Specifically, the labels ② and ③, which consist of ellipse and rhombus, perform a little bit better than the ellipse labels. On the whole, the rhombus labels have the best performance compared to the other three label shapes. It indicates that our rhombuses labels can debase interference of the error-prone sample and get a more noiseless classification. 

## 5. Conclusions

In this work, we propose a Siamese network tracker called TA-Siam, which combines the template adjustment module, novel classification rhombus labels, and anchor-free regression. Our plug-and-play template adjustment module adaptively adjusts template features to overcome the model drifts and tracking failure in complex tracking scenes, such as illumination variation, rotation, occlusion, out of view, and more. While in classification label selection, we proposed unique rhombus labels to markedly decrease the impact of error-prone samples compared with ellipse labels and rectangle labels. In the anchor-free regression, we implement the distance-IOU loss function to constrain the distance of center and corner. It makes the framework obtain faster convergence and efficient and better training effect. The experiments on six visual tracking benchmarks demonstrate that our TA-Siam achieves state-of-the-art performance with a fast average speed (45 FPS). In our future work, we will study the adaptive adjustments to obtain a more robust template. Besides, a more approximate and convergent regression loss function is also an important point.

## Figures and Tables

**Table 1:** Performance comparisons on VOT2016 benchmark.

	SiamRPN	SPM	ROAM	SPS	SiamRPN++	UpdateNet	SiamBAN	SiamAttn	Ours
EAO↑	0.337	0.434	0.441	0.459	0.478	0.481	0.494	0.537	0.555
A↑	0.578	0.620	0.599	0.625	0.637	0.610	0.632	0.680	0.628
R↓	0.312	0.210	0.174	0.158	0.177	0.210	0.158	0.140	0.107

EAO, Expected Average Overlap; A, accuracy; R, robustness. ↑ indicates that the larger the value, the better the performance. While ↓ indicates the smaller the value, the better the performance.

**Table 2:** Performance comparisons on VOT2018 benchmark.

	FCOS-MAML	ATOM	SiamRPN++	SiamFC++	SPS	PrDiMP	SiamBAN	Retina-MAML	Ours
EAO↑	0.392	0.400	0.414	0.426	0.434	0.442	0.452	0.452	0.469
A↑	0.635	0.590	0.600	0.587	0.612	0.618	0.597	0.604	0.592
R↓	0.220	0.203	0.234	0.183	0.169	0.165	0.178	0.159	0.155

The “FCOS-MAML” and “Retina-MAML” indicate MAML [39] trackers based on detectors RetinaNet [29] and FCOS [26] respectively. ↑ indicates that the larger the value, the better the performance. While ↓ indicates the smaller the value, the better the performance.

**Table 3:** Performance comparisons on the GOT-10k benchmark.

	SiamDW	THOR	ROAM	SPM	SiamRPN++	ATOM	SiamCAR	SiamFC++	D3S	Ours
AO	0.416	0.447	0.465	0.513	0.517	0.556	0.569	0.595	0.597	0.608
SR_0.5_	0.475	0.538	0.532	0.593	0.616	0.634	0.670	0.695	0.676	0.731
SR_0.75_	0.144	0.204	0.236	0.359	0.325	0.402	0.415	0.479	0.462	0.455

AO, Average Overlap; SR, Success Rate.

**Table 4:** Components analysis of TA-Siam. “TAM” represents template adjustment module, “DIOU” means distance-IOU loss.

#Num	Components	EAO↑	R (Failure Rate)↓
①	baseline	0.494	0.158
②	+DIOU	0.505	0.149
③	+TAM	0.538	0.121
④	+TAM+DIOU	0.555	0.107

↑ indicates that the larger the value, the better the performance. While ↓ indicates the smaller the value, the better the performance.

**Table 5:** Analysis of the impact of different label shapes.

#Num	Label Shapes	AO↑
①	Ellipses	0.575
②	Rhombus + Ellipse	0.581
③	Ellipse + Rhombus	0.577
④	Rhombuses	0.608

↑ indicates that the larger the value, the better the performance.

## Data Availability

Most data generated of analyzed during this study are included in the submitted article. Raw data and derived data supporting the findings of the study are also available from the first author upon request.
